# Muscle strength and regional lean body mass influence on mineral bone health in young male adults

**DOI:** 10.1371/journal.pone.0191769

**Published:** 2018-01-25

**Authors:** Bianca Rosa Guimarães, Luciana Duarte Pimenta, Danilo Alexandre Massini, Daniel dos Santos, Leandro Oliveira da Cruz Siqueira, Astor Reis Simionato, Luiz Gustavo Almeida dos Santos, Cassiano Merussi Neiva, Dalton Muller Pessôa Filho

**Affiliations:** 1 Departament of Physiotherapy, University of Alfenas (Unifenas), Divinópolis, Minas Gerais, Brazil; 2 Postgraduate Program in Health Promotion, University of Franca (Unifran), Franca, São Paulo, Brazil; 3 Postgraduate Program in Human Development and Technology, Bioscience Institute of Biosciences, São Paulo State University (UNESP), Rio Claro, São Paulo, Brazil; 4 Department of Physical Education, College of Sciences, São Paulo State University (UNESP), Bauru, São Paulo, Brazil; Charles P. Darby Children’s Research Institute, UNITED STATES

## Abstract

The relationship between muscle strength and bone mineral content (BMC) and bone mineral density (BMD) is supposed from the assumption of the mechanical stress influence on bone tissue metabolism. However, the direct relationship is not well established in younger men, since the enhancement of force able to produce effective changes in bone health, still needs to be further studied. This study aimed to analyze the influence of muscle strength on BMC and BMD in undergraduate students. Thirty six men (24.9 ± 8.6 y/o) were evaluated for regional and whole-body composition by dual energy X-ray absorptiometry (DXA). One repetition maximum tests (1RM) were assessed on flat bench-press (BP), lat-pull down (LPD), leg-curl (LC), knee extension (KE), and leg-press 45° (LP45) exercises. Linear regression modelled the relationships of BMD and BMC to the regional body composition and 1RM values. Measurements of dispersion and error (R^2^_adj_ and standard error of estimate (SEE)) were tested, setting ρ at ≤0.05. The BMD mean value for whole-body was 1.12±0.09 g/cm^2^ and BMC attained 2477.9 ± 379.2 g. The regional lean mass (LM) in upper-limbs (UL) (= 6.80±1.21 kg) was related to BMC and BMD for UL (R^2^_adj_ = 0.74, p<0.01, SEE = 31.0 g and R^2^_adj_ = 0.63, SEE = 0.08 g/cm^2^), and LM in lower-limbs (LL) (= 19.13±2.50 kg) related to BMC and BMD for LL (R^2^_adj_ = 0.68, p<0,01, SEE = 99.3 g and R^2^_adj_ = 0.50, SEE = 0.20 g/cm^2^). The 1RM in BP was related to BMD (R^2^_adj_ = 0.51, SEE = 0.09 g/cm^2^), which was the strongest relationship among values of 1RM for men; but, 1RM on LPD was related to BMC (R^2^_adj_ = 0.47, p<0.01, SEE = 44.6 g), and LC was related to both BMC (R^2^_adj_ = 0.36, p<0.01, SEE = 142.0 g) and BMD (R^2^_adj_ = 0.29, p<0.01, SEE = 0.23 g/cm^2^). Hence, 1RM for multi-joint exercises is relevant to BMC and BMD in young men, strengthening the relationship between force and LM, and suggesting both to parametrizes bone mineral health.

## Introduction

The mineral content of bone tissue (BMC) is a component of the body composition, providing the fat-free mass (FFM) when associated with the lean mass (LM) (musculature and viscera) [1, 2, 3, 4]. Bone mineral density (BMD) reflects the integrity of the bone tissue, indicating its capacity of structural remodeling and, therefore, is an index of the risk for pathologies and lesions associated with the tissue [1, 5]. Actually, the loss of bone mineral content hasn’t only been associated to the aging process or hormonal dysfunctions, but also to the decline of fat mass (FM) and FFM from dietary programs for weight loss and to a sedentary lifestyle [1, 6, 7].

It has been observed that the reduction in BMD is positively associated with age, evidencing 0.6%, 1.1% and 2.1% of loss over the range between 60–69, 70–79 and ≥ 80 years old, respectively [5]. These rates of reductions often culminate with osteoporosis, but tend to be minimized by regular physical activities and the maintenance of body fat and fat-free content in healthy patterns [3, 6, 7]. Having analyzed the association between aging and the reduction of physical activities, BMC and FFM, Proctor et al. [7] reported that from 20 to 80 years of age there is a tendency of physical activity reduction, ranging between 34–38% both for women and men, in association to the reductions of FFM (18–17%) and FM (16–30%). These authors also observed elevated and significant correlations between FFM and FM for men (r = 0.77) and women (r = 0.74). The study by Lee et al. [1] contributed to supporting this association, concluding that FFM is a significant and independent determinant to the whole-body and regional bone mineral mass, reporting low but significant coefficients of variance (R^2^ < 0.5, p < 0,01) to the association between appendicular mass (Kg) and the total pelvic and forearm BMD. These associations among body composition and mineral bone mass were also observed in the study of Makovey et al. [3] with both genders, from different age ranges. According to these authors, not only FFM but also body FM positively influence BMC with explanatory potential of 52% between the FFM and BMC and of 20% between the body FM and BMC. The explanations to these associations are the neuromuscular system’s integrity as a mechanical factor, which acts as a regulator of osteogenic activity, and the fat’s influence over estrogen secretion as a humoral factor on the regulation of the osteoblast activities [1, 3].

Generally, physical activity including resistance or endurance exercises tends to promote alterations in BMD and BMC by mechanical stress on bones, and weight training tends to be the physical activity with the highest potential to stimulate changes or maintenance of BMD and BMC with aging [6]. The role of aerobic exercises, when performed at moderate intensity (such as walking), is to induce changes of BMD by the increase in gravitational loading on the skeleton [8, 9, 10]. However the results on the effectiveness of aerobic exercises speculate that higher intensity exercise practice would increase the benefits related with prevention/treatment of the disturbances associated with BMD reduction, even though there are no conclusive studies about the exercise rate that is more suitable to attain this goal. Walking exercises with long-term training protocols (> 20 weeks), either combined or not with other activities (such as steps or rowing) tend to increase BMD of the femoral and lumbar region (~ 2 to 5%), or at least avoid reductions when compared to the non-exercising counterparts (control groups), showing reductions of over 7% [8, 9, 10, 11].

On the other hand, strength training has protocols with recognized influence on the improvements of BMD, which includes heavy loads, 2–3 sets for each exercise, designed with a frequency of 3 times per week for 4–6 months [12, 13, 14]. Long-term training protocols (>12 months), with load intensity between 50–80% of one repetition maximum (1RM) for upper- (UL) and lower-limbs (LL) increases BMD up to 3.8%, or significantly prevent reductions (~2.5%), when compared to non-exercising individuals in the control group [12, 13, 14, 15]. Collectively, these studies suggested design protocols with loads of 12 to 15 repetitions to maximum or 70–80% of 1RM.

The relationship between muscle strength and BMD (and BMC) is supposed from the assumption of the mechanical stress influence on bone tissue metabolism. However, healthy bone mineral is *a priori* assumed to have no disturbance among young people, and changes in muscle strength are not able to discriminate more or less healthier mineralized bone. Nonetheless, the relationship between muscle strength and FFM (whole-body or regional) is well-stated, which leads us to presume a possible tendency of the changes of muscle strength to be positively related with changes of BMD (or BMC), based on the assumption that the relationship of FFM to BMC or BMD is well established. Thus, this study aimed to analyze the influence of muscle strength on bone mineral health (BMD and BMC) among young adult males, searching for relationships that could establish causal effect either on regional or whole-body BMD or BMC, and be able to parameterize the healthy state or the risk of disturbance.

## Material and methods

### Subjects

The participants (n = 36) presented the following characteristics: 24.9 ± 8.6 years old, 175.2 ± 5.1 cm height and 71.2 ± 12.6 kg body weight. All participants were men and received verbal orientation on the procedures and signed a self-informed consent which authorized participation in this research, according to the principles expressed in the Declaration of Helsinki. When the participant was under 18 years old, the consent form was also signed to his/her parents. This research was approved to the Ethics Committee of São Paulo State University (UNESP) (CAEE: 70076317.1.0000.5398).

### Study design

Initially, the individuals were submitted to regional and whole-body composition by dual energy X-ray absorptiometry (DXA). After this the 1RM test was applied. The 1RM represented muscle strength in five exercises (two for UL and three for LL) tested and re-tested 24h apart. The procedures did not exceed 45 min duration per day, and all experimental trials were concluded after a week for each participant. The individuals were instructed to avoid training with heavy loads as well as alcoholic drinks and coffee (caffeine) for 24 h prior to the tests. The protocol was registered on Open Access Repository of Science Methods platform under DOI: https://dx.doi.org/10.17504/protocols.io.kxacxie.

### Body composition

DXA (modelo Hologic^®^, QDR Descoberta Wi^®^) was used to obtain the regional and whole-body composition, as suggested by Wang et al. [16] and Kohrt [17]. The software (Hologic APEX^®^) yields absolute (in grams) values of FM, FFM (which includes measures of BMC, in grams), BMD (in grams/cm^2^), LM (which does not include BMC), and total mass for whole-body and regional references (head, trunk, left arm, right arm, left leg and right leg) [4]. The UL and LL composition were further considered, which were obtained from a simple algebraic sum of corresponding regional reference. The equipment was calibrated according to the manufacturer’s recommendations and all analyses were performed by an experienced technician. According to Nana et al.[18], the participants should wear light clothing, no shoes or have any metallic objects attached to the body and clothes. The participants were positioned in the supine position on a flat table until the end of the checking. Their feet remained close together and their arms were placed parallel to the trunk. Lines were adjusted and aligned by the same technician through specific anatomic points determined by the software.

### Strength measure

The 1RM tests were performed on: (a) flat bench-press (BP), (b) lat pull-down (LPD), (c) knee-extension (KE), (d) leg-curl (LC), and (e) leg press 45° (LP45). All tests were performed after a 15 min warm-up (static stretching, and aerobic exercise on a bike or running with low workload/velocity intensity). The 1RM test protocol followed the recommendations of Mayhew et al. [19] and Baechle and Earle [20], being (1) a specific warm-up preceded the test, and included repetitions performed with light intensity loads avoiding concentric failure; (2) initial attempt for one maximum repetition was performed with load related rating scores for UL and LL strength, according to age, gender and body-weight; (3) the participants performed at least three attempts with 3 min resting between each, increasing or decreasing the initial lifted load from 1.1 to 4.5 kg, according to the level of difficulty of the first attempt. The highest weight (in kg) successfully lifted was the reference value of 1RM. The 1RM load was submitted to a confirmatory test, consisting of two additional attempts performed 24 h apart. For the confirmatory test, the load at 1RM was fractioned into percentages of 90, 95, 100, 105 and 110%, which were randomly chosen and lifted with a 3 min rest between them. It was mandatory to try one lift with a load above 1RM if the first load chosen was 100% 1RM or less. The participants were instructed to perform the movements with the adequate technique, following standardized recommendations for the movement [20].

### Statistics

Data were presented by Mean ± SD and its corresponding range (minimum and maximum). Normality was verified by the Kolmogorov-Smirnov’s test. The linear correlation coefficient (Pearson’s “r”) was applied to the analysis of the relationships from values of BMD and BMC (as dependent factors) to the regional and whole-body composition and 1RM for BP, LPD, KE, LC and LP45 (as independent factors). The dispersion and variability for the deterministic relationship between dependent and independent variables were measured by the coefficient of variance adjusted to the sample (R^2^_Adj_), and the standard error of estimate (SEE). The stepwise method was applied to regression analysis, and the significance level was set at p≤0.05. All procedures were performed by SPSS 15 (Statistical Package for Social Sciences, Inc., USA).

The sample power for the correlations between dependent and independent variables was determined considering the sample size (Men = 36). The entry parameters were: (a) the Pearson “r” coefficient; (b) Zα = 1.96 to a security index of α = 0.05; and (c) Z_1_-β = 1.282 for an expected sample power of 80% (β = 0.20), according to Diaz and Fernandez [21].

$$Z_{1-\beta }=\sqrt{n-3}\frac{1}{2}ln\left(\frac{1+r}{1-r}\right)-Z_{1-\frac{\alpha }{2}}$$

In addition to the sample power, magnitude-based inference analysis was applied to test the chances of the true magnitude of an effect to be substantially positive and negative, and negligible or trivial (with odds ratio of 66 to ensure that >25% chance of benefit and <0.5% chance of harm means a decisively useful effect). The chances were given qualitatively from threshold values, according to the scale: <1% = most unlikely; 1%–5% = very unlikely; 5%–25% = unlikely; 25%–75% = possibly; 75%–95% = likely; 95%–99.5% = very likely; and >100% = most likely. This procedure ensures that the study with the sampling distribution of z [= 0.5 × ln × ((1+r)/(1-r))] would be reproduced normally with variance [= 1/(n-3)] [22].

## Results

The regional and whole-body values for BMC and BMD are presented in Table 1. The “*T-score*” (= -0.90) classified BMD as normal, considering the standard reference for young adults as “*T-score*” = -1.00 for both genders. The percentage of whole-body FM was 18.0±6.2%, classifying obesity as normal. Table 2 shows values of Pearson’s coefficient to the correlation observed for LM to BMC and BMD either for regional or whole-body compositions. Thus, the values of LM for whole-body (55.41±7.64 kg), UL (6.80±1.21 kg), trunk (25.88±4.14 kg) and LL (19.13±2.50 kg) all presented moderate to high and significant correlations to BMC and BMD, but regional values for UL and LL coefficients were larger than those for whole-body.

**Table 1:** Average±SD values to whole-body and regional BMC and BMD of subjects (N = 36).

		Mean ± SD	Minimum–Maximum
BMC (g)	Trunk	659.7 ± 131.5	443.1–1048.2 g
	Upper-limbs	348.8 ± 60.4	238.1–516.3 g
	Lower-limbs	949.8 ± 175.1	645.8–1320.4 g
	S. Thorax	120.2 ± 24.7	78.4–210.5 g
	S. Lumbar	66.4 ± 15.1	41.8–102.7 g
	Pelvis	268.5 ± 66.9	177.2–427.6 g
	Whole-body	2477.9 ± 379.2	1754.4–3380.4 g
BMD (g/cm^2^)	Trunk	4.51 ± 0.51	3.75–5.78 g/cm^2^
	Upper-limbs	1.57 ± 0.14	1.36–1.96 g/cm^2^
	Lower-limbs	2.41 ± 0.27	2.01–3.08 g/cm^2^
	S. Thorax	0.85 ± 0.10	0.68–1.14 g/cm^2^
	S. Lumbar	1.11 ± 0.16	0.85–1.55 g/cm^2^
	Pelvis	1.16 ± 0.17	0.89–1.66 g/cm^2^
	Whole-body	1.12 ± 0.09	0.97–1.40 g/cm^2^

**Table 2:** Pearson’s coefficients for the correlations between whole-body and regional lean mass to BMC and BMD.

		Lean mass (g) (N = 36)			
		Whole-body	Trunk	Upper-limbs	Lower-limbs
BMC (g)	Whole-body	0.815**	0.756**	0.816**	0.819**
	S. Thorax	0.728**	0.619**	0.718**	0.726**
	S. Lumbar	0.615**	0.551**	0.628**	0.642**
	Pelvis	0.662**	0.518**	0.715**	0.721**
	Trunk	0.816**	0.679**	0.819**	0.825**
	Upper-limbs	0.838**	0.781**	0.862**	0.744**
	Lower-limbs	0.773**	0.698**	0.812**	0.829**
BMD (g/cm^2^)	Whole-body	0.629**	0.623**	0.652**	0.640**
	S. Thorax	0.631**	0.510**	0.660**	0.672**
	S. Lumbar	0.481**	0.402*	0.563**	0.577**
	Pelvis	0.595**	0.455**	0.594**	0.610**
	Trunk	0.501**	0.593**	0.586**	0.695**
	Upper-limbs	0.756**	0.734**	0.797**	0.653**
	Lower-limbs	0.466**	0.658**	0.595**	0.704**

Obs.: correlation with significance at

*p≤0.05 e

**p≤0.01.

**Fig 1:**
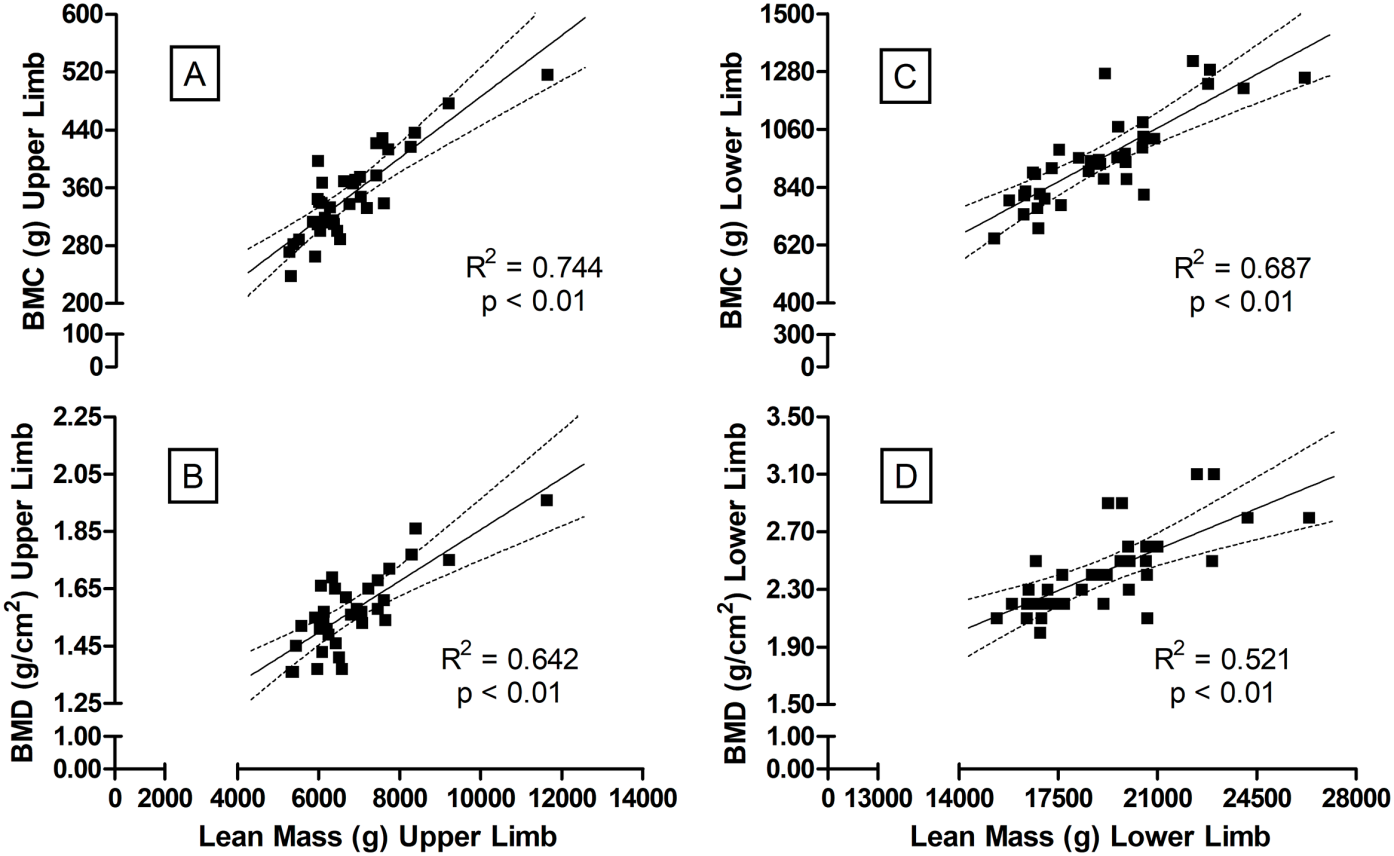
Regression analysis between lean mass in upper-limbs (Panels A and B) and lower-limbs (Panels C and D) with BMC and BMD. BMC and BMD refers to bone mineral content and density, respectively. N = 36.

The power of LM to determine changes in BMC and BMD is illustrated in Fig 1. From the relationships observed in Fig 1 a “regional specificity” for the association between values of LM and BMC/BMD can be noted. The LM in UL is related to BMC in UL (Fig 1, Panel A) with R^2^_adj_ (= 0.74, p<0.01), SEE (= 31.0 g) and sample power (= 100%), which is considered “most likely to be substantially positive”. With the same sample power (= 99.9–100%) and qualitative scale for the chances to be a “true effect” (most likely to be substantially positive), the BMD in LM in UL was related to BMD (Fig 1, Panel B) with R^2^_adj_ (= 0.63, p<0,01) and SEE (= 0.08 g/cm^2^), as well as the association between LM in LL to BMC (Fig 1, Panel C: R^2^_adj_ = 0.68, p<0,01 and SEE = 99.3 g) and BMD (Fig 1, Panel D: R^2^_adj_ = 0.50, p<0,01 and SEE = 0.20 g/cm^2^) in LL.

The values of muscle strength from the 1RM test on BP (59.8 ± 15.6 kg), LPD (62.0 ± 16.1 kg), LC (71.7 ± 14.4 kg), LE (91.4 ± 29.9 kg), and LP45 (256.6 ± 57.0 kg) were all related to the whole-body values of BMC and BMD, as well as related to the regional values of BMC/BMD for UL (Table 3). From Table 3 it can also be observed that the correlation coefficients are significant between muscle strength and BMC/BMD regional values for LL, trunk and pelvis (with the exception of muscle strength in the leg extension exercise).

**Table 3:** Pearson’s coefficient for the correlation between muscle strength and whole-body and regional values of BMC/BMD.

		Strength (kg) from 1RM test (N = 36)				
		Bench Press	Lat Pull Down	Leg Curl	Leg Extension	Leg Press 45°
BMC (g)	Whole-body	0.469**	0.519**	0.580**	0.363*	0.463**
	S. Thorax	*ns*	*ns*	*ns*	*ns*	0.346*
	S. Lumbar	*ns*	*ns*	*ns*	*ns*	*ns*
	Pelvis	0.355*	*ns*	0.471**	*ns*	0.462**
	Trunk	0.465**	0.397*	0.458**	*ns*	0.487**
	Upper-limbs	0.565**	0.686**	0.536**	0.493**	0.432**
	Lower-limbs	0.370*	0.459**	0.600**	*ns*	0.423*
BMD (g/cm^2^)	Whole-body	0.492**	0.470**	0.564**	*ns*	0.458**
	S. Thorax	*ns*	*ns*	*ns*	*ns*	*ns*
	S. Lumbar	*ns*	*ns*	*ns*	*ns*	*ns*
	Pelvis	0.404*	*ns*	0.418*	*ns*	0.401*
	Trunk	0.451**	0.365*	0.447**	*ns*	0.423*
	Upper-limbs	0.723**	0.722**	0.527**	0.430**	0.600**
	Lower-limbs	0.465**	0.457**	0.556**	*ns*	0.461**

The term “ns” is “not significant” for correlation with p>0.05. Obs.: correlation with significance at

*p≤0.05 and

**p≤0.01.

**Fig 2:**
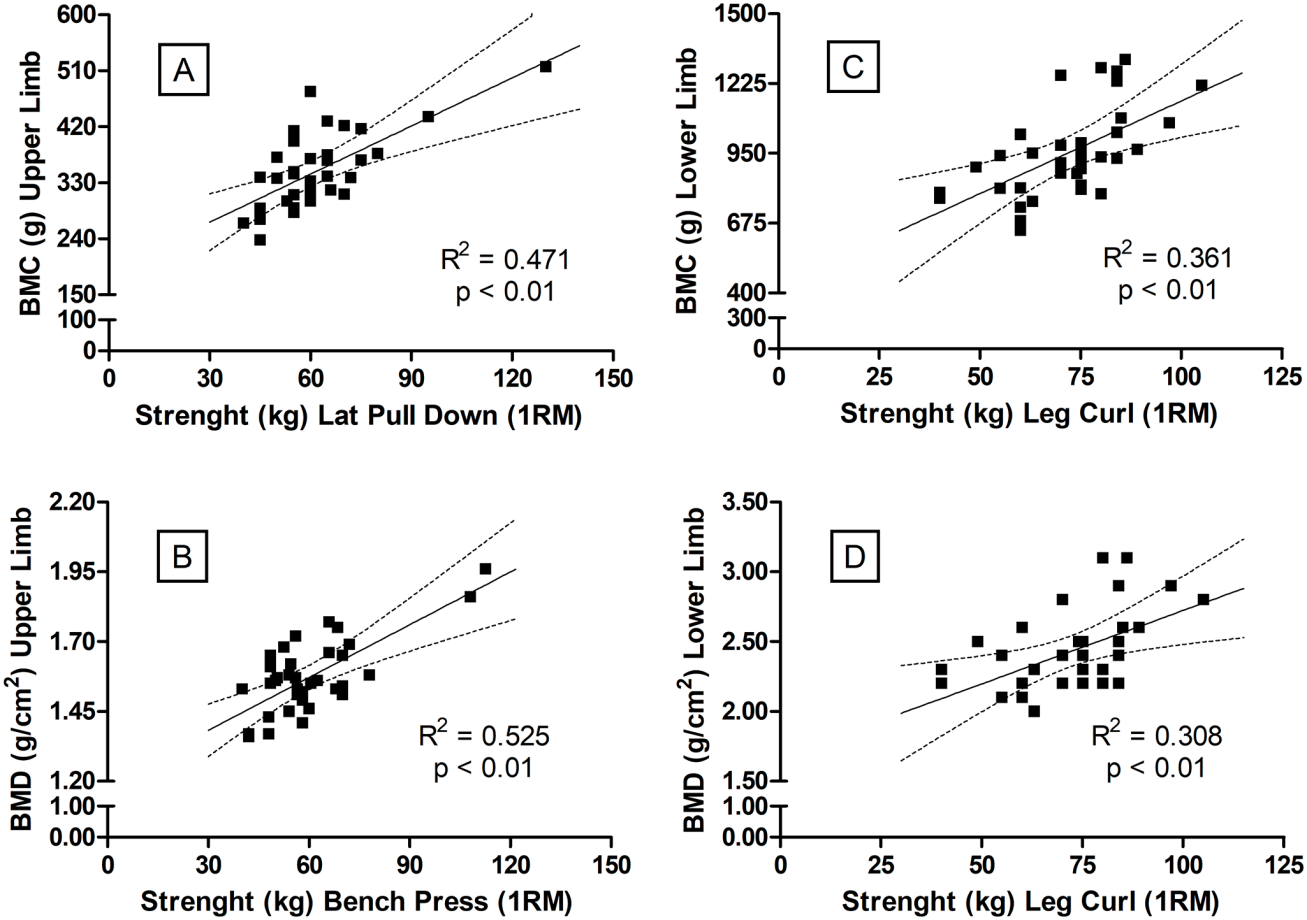
Regression analysis between strength (1RM) on resistance exercises for upper- (Panels A and B) and lower-limbs (Panels C and D) with BMC and BMD. BMC and BMD refers to bone mineral content and density, respectively. N = 36.

The level of muscle strength association to the changes of BMC and BMD is shown in Fig 2. From Fig 2 (Panel A and B) the specificity trends of regional relationship can also be noted. The maximum force (1RM) on LPD (*i*.*e*. the ability of UL to apply force in pulling actions) was related to BMC (Panel A) with R^2^_adj_ (= 0.45, p<0.01), SEE (= 44.6 g) and sample power (= 99.6%), which is considered “most likely to be substantially positive”. The BMC changes are also related to BP (*i*.*e*. the ability of UL to apply force in pushing actions) (Panel B) with R^2^_adj_ (= 0.51, p<0.01), SEE (= 0.09 g/cm^2^) and sample power (= 99.9%), which was considered a “true effect” (most likely to be substantially positive). The same quality of effect was observed for the relationships between LC (*i*.*e*. the ability of the thigh to apply force in pulling actions) and BMC (Panel C) with R^2^_adj_ (= 0.34, p<0.01), SEE (= 142.0 g) and sample power (= 95.8%); and between LC and BMD (Panel D) with R^2^_adj_ (= 0.29, p<0.01), SEE (= 0.23 g/cm^2^) and sample power (= 89.7%).

## Discussion

The major determinants of the loss of bone mineral mass are aging and a decline in physical activity [5, 6]. However, an elderly and sedentary lifestyle also compromise lean body mass, which represents the skeletal muscle [23]. However, the results from the present study demonstrated that variations in BMC and BMD among young men are associated with both UL and LL muscle strength and regional and whole-body LM. These results are consistent with the fact that LM and BMD/BMC are strongly related variables, regardless of gender and age, especially among persons younger than 50 years [3]. Moreover, the results showed that muscle strength also correlates to BMC/BMD with magnitude similar (or only slightly lower) to the association that the LM parameter of regional and whole-body composition demonstrated to have had to BMC/BMD. This tendency of association between strength capacity and BMC/BMD has been little evidenced, although muscle strength is consistently reported as indicative of muscle mass and functional quality among people of any gender and age [5]. This means that any exercise condition (but specifically resistance exercise) which is able to combine improvements of muscle strength and muscle mass as the result of practice routine, is also expected to ensure ideal conditions to stimulate the osteogenic process, either by promoting appropriate mechanical stress during the practice or by reducing propensity to accumulate fat while engaged in the exercise program planned with this kind of practice [1, 5, 6, 23, 24].

In turn, lean body mass has presented relationships to BMC/BMD, especially among elderly men [3, 6]. In part, this relationship can be explained by the fact that BMC/BMD declines more sharply with the aging process in women than men, which is also associated to reductions in LM and level of physical activity. In the study by Proctor et al. [6], this reduction was quantified. For these authors, BMC/BMD declines by 30% between 20 and 80 years of age in women, but only 16% in this same age range among men, whereas the reduction of LM is 18% and 17% and physical activity is 34% and 38%, respectively, among women and men in the same age range. That is, men tend to maintain a more stable ratio between LM and mineral mass in the composition of FFM when compared to women [24]. However, men’s decrements of mobility and vitality with aging are more susceptible to the reductions in BMC/BMD and LM, constraining the maintenance of physical activity levels. This evidence is aligned to the findings of Horber et al. [24] who observed a sex-specificity of how body composition and muscle metabolism change with age. These authors noted that the loss of LM among men is associated with the accumulation of fat in the trunk and UL, as well as promoting a reduction of fat oxidation in muscle. This trend supported the understanding of the influence of reducing LM on physical fitness levels among men, but also suggests the importance of regional analysis of morphological and functional characteristics, since they may be also associated to the sex-related preferences for exercises engaging specific body regions [25].

However, regional LM is still poorly explored as an independent factor influencing BMC/BMD [15, 23, 25], perhaps because the experimental designs involved specific measures of BMD/BMC (hip, vertebrae and pelvis), as well as the studies often engaging elderly participants with a diagnosis of bone mineral integrity disorders (Osteopenia and Osteoporosis) [6, 23, 25]. Thus, the present study supports the positive association between regional LM and regional and whole-body BMC/BMD, adding the occurrence of a local trend that makes the association more specific among variables of the same body region. Thus, if some studies are emphatic in assuming a dubious posture about which body composition parameter (LM, FM or both) influences the integrity of bone mineral mass, among men and women with aging [3], the results from the present study reinforce that the regional LM of the legs and arms (right and left) tends to emphasize the role of regional composition as an independent factor influencing bone mineral integrity, which is potentially greater than that reported for whole-body FM.

In the present study, the assessment of muscle maximum force in resistive exercises also presented potential to indicate variations in regional and whole-body BMC/BMD in young men. This observation is aligned to the information indicating that muscle strength is able to parameterize BMC/BMD alterations such as regional LM (legs or arms) or whole-body mass [26]. On the other hand, there are studies not in agreement with this perspective, indicating that the increase of muscular strength is not related to the increase of muscle mass, nor is muscle strength a potential parameter of the variations in BMC/BMD [27]. Thus, the results of the present study contributed to the information that muscular strength is likely to be an index of bone mineral integrity, either to the direct influence on BMC/BMD or indirectly by influencing LM and its functional capacity.

Additionally, muscle strength in BP, knee flexion and extension are considered exercises that engage a large amount of muscular mass to be utilized [28], and therefore have both regional and whole-body influence on BMC/BMD. Despite the modest predictive potential, which is no higher than that presented to the regional and whole-body LM, these results are partially in accordance with data observed by Hughes et al. [29] about the lack of association between BMD and muscle strength in single-joint exercises, suggesting whole-body LM as the single strongest estimator. However, the present results are in agreement with the observations of Lee et al. [1] recommending the development of muscle strength to reduce the risks of osteoporosis, since it is related to whole-body lean and bone mass of the hip (region with the highest occurrence of osteopenia). However, our results indicated that regional BMC/BMD in the pelvis (which includes the pelvic bones and femoral head) and trunk (which includes the thoracic and lumbar vertebrae) [30] also showed significant associations to muscle strength in single-joint exercises (knee flexion and extension) and multi-joint exercises (BP and LP45). Although these correlations were not higher than those presented between regional and whole-body lean body mass with pelvis BMC, such results extend the findings of Lee et al., [1] suggesting muscle strength either in global or local exercises as an appropriated index to monitor the risk of osteoporosis in the pelvic and trunk bone sites.

However, our analysis did not approach specific sites (hip and lower spine) when assessing densitometry and establishing relationships to muscle strength and regional and whole-body composition. Neither was considered the regional and whole-body FM influence on BMD [31, 32]. These are limitations of the present study, since the analysis of specific sites would make the potential of the results more elucidative for practical diagnosis, whereas a possible correlation between BMC/BMD and FM (regional or whole-body) could reduce the deterministic potential of LM mass, which was observed in the present study among young men.

Despite bone turnover markers (BTMs) not being within the scope of the present study, it is important to consider them due their role as a reliable measurement of resorption/formation balance of the bone mineral mass. The BTMs include serum osteocalcin and alkaline phosphatase, dictating the increase in mineral mass of the bone, and pyridinoline, urine/serum C-terminal telopeptide (CTX), and urine N-terminal telopeptide (NTX), which otherwise promote bone mass loss [33, 34]. Such serum and urine BTMs are clinically relevant to the diagnosis of osteopenia and osteoporosis, since BTMs relate to parameters having detrimental effects on bone metabolism. Therefore, long-term changes in the BMD able to reduce values below that proposed for osteoporosis (0.708 g/cm3), are associated to the increased urine NTX levels (- 0.68, p = = 0.002) and alkaline phosphatase (- 0.49, p = 0.04), both signalizing accelerated bone resorption and formation [35]. However, Lenora et al. [33] observed no substantial difference between the ability of the several BTMs to predict BMD changes in the arms, legs, total body, total hip or femoral neck region and lumbar spine, although the authors considered all associations weak. Despite such inconsistency in the association of BMT markers to BMD, there are reports postulating that age-dependent attenuation of GH and IGF-I are associated with femoral bone loss and hormone steroids, such as androgen and estrogen, interacting with GH and IGF-1, optimizing bone mass growth and maintenance [36]. Furthermore, the circulating level of IGF-1 is considered an independent predictor of total BMC in healthy elderly women, and a determinant of hip BMD in young men (age under 60 years) [36].

Other aspects affecting muscle and bone development are exercise and nutrition. For example, bone formation is lower in the obese subjects suggesting that the serum BTM rate is suppressed in this population, probably because Leptin releases from adipocytes correlates inversely with all BTMs [37]. However, hyperglycemia induces a low turnover of bone with osteoblast dysfunction and suppresses serum osteocalcin levels (R = 0.133 p = 0.0467), showing that the serum osteocalcin level was negatively correlated with the percentage of body fat (%Fat) [34]. Indeed, circulating levels of IGF-1 are associated with endurance exercise indexes, highlighting the importance of a healthy lifestyle for bone osteogenic signalization [36]. Taking collectively, exercise and nutrition have a pronounced effect on body composition in men and women, but the associations between body composition and bone mineral variables (BMC and BMD) are related to gender and age differences. From the study of Makovey et al. [3] stronger relationships of whole-body lean mass with whole-body BMC and regional measurements of BMD for all age groups (< 50 and ≥ 50 years) in both genders was observed, which was not only greater than the associations of hip BMD with whole-body fat mass in females under 50 years. The aforementioned authors have concluded that fat mass and lean mass would probably increase BMD by increasing mechanical loading on the skeleton, but in women fat mass exerted an additional effect possibly mediated by the serum leptin level. In the present study, our results added that the association between bone mineral measures and regional lean mass distribution is stronger in younger age groups than whole-body composition. Also, it was revealed that muscle strength plays not merely a coadjutant role in bone mineral mass, whereas it is still unrevealed whether this association is supported for the higher concentrations of BTMs in males than females [37], or by the influence of muscle and strength on circulating levels of androgens in men [36].

## Conclusion

In the present study, the results confirmed regional LM as an influential factor on bone mineral integrity, in magnitude similar to LM for whole-body, suggesting that the muscular strength capacity is also determinant on regional and whole-body BMC/BMD. Thus, the development of muscular strength is repeatedly important for the maintenance of LM in young people and positively affecting bone mass. It is therefore recommended that resistance exercise takes part of the exercise planning and that the prescription matches the goal of regional LM enhancement in UL and LL. In addition, training is recommended with the aim at reducing fat accumulation, as well as ensuring functional independence. We recommended that future studies should explore whether the caloric cost of different training routines (resistance exercise, cardiorespiratory endurance and/or combinations) would give a more conclusive parameter to define the intensity of exercise that would be most effective to promote morphological changes of lean tissue. Additionally, it is recommended that future studies explore specific body sites with high incidence of osteoporosis.

## Supporting information

S1 File HOMENS_DEXA-FORÇA. (SAV)
